# Friction Stir Welding of 2205 Duplex Stainless Steel: Feasibility of Butt Joint Groove Filling in Comparison to Gas Tungsten Arc Welding

**DOI:** 10.3390/ma14164597

**Published:** 2021-08-16

**Authors:** Mohamed M. Z. Ahmed, Khaled A. Abdelazem, Mohamed M. El-Sayed Seleman, Bandar Alzahrani, Kamel Touileb, Nabil Jouini, Ismail G. El-Batanony, Hussein M. Abd El-Aziz

**Affiliations:** 1 Mechanical Engineering Department, College of Engineering at Al Kharj, Prince Sattam Bin Abdulaziz University, Al Kharj 16273, Saudi Arabia; ba.alzahrani@psau.edu.sa (B.A.); k.touileb@psau.edu.sa (K.T.); n.jouini@psau.edu.sa (N.J.); 2 Department of Metallurgical and Materials Engineering, Faculty of Petroleum and Mining Engineering, Suez University, Suez 43512, Egypt; Mohamed.elnagar@suezuniv.edu.eg; 3 PETROJET Company, Cairo 11835, Egypt; kabdelazem@yahoo.com; 4 Mechanical Engineering Department, Faculty of Engineering, Al-Azhar University, Cairo 11651, Egypt; ismailghazy2017@azhar.edu.eg; 5 Laboratoire de Mécanique, Matériaux et Procédés (LR99ES05), École Nationale Supérieure d’Ingénieurs de Tunis, Université de Tunis, Tunis 1008, Tunisia; 6 Mining and Petroleum Engineering Department, Faculty of Engineering, Al-Azhar University, Cairo 11651, Egypt; dr_eng_hussein@yahoo.com

**Keywords:** groove joint design, friction stir welding, gas tungsten arc welding, 2205 DSS, mechanical properties

## Abstract

This work investigates the feasibility of using friction stir welding (FSW) process as a groove filling welding technique to weld duplex stainless steel (DSS) that is extensively used by petroleum service companies and marine industries. For the FSW experiments, three different groove geometries without root gap were designed and machined in a DSS plates 6.5 mm thick. FSW were carried out to produce butt-joints at a constant tool rotation rate of 300 rpm, traverse welding speed of 25 mm/min, and tilt angle of 3^o^ using tungsten carbide (WC) tool. For comparison, the same DSS plates were welded using gas tungsten arc welding (GTAW). The produced joints were evaluated and characterized using radiographic inspection, optical microscopy, and hardness and tensile testing. Electron back scattering diffraction (EBSD) was used to examine the grain structure and phases before and after FSW. The initial results indicate that FSW were used successfully to weld DSS joints with different groove designs with defect-free joints produced using the 60° V-shape groove with a 2 mm root face without root gap. This friction stir welded (FSWed) joint was further investigated and compared with the GTAW joint. The FSWed joint microstructure mainly consists of α and γ with significant grain refining; the GTWA weld contains different austenitic-phase (γ) morphologies such as grain boundary austenite (GBA), intragranular austenite precipitates (IGA), and Widmanstätten austenite (WA) besides the ferrite phase (α) in the weld zone (WZ) due to the used high heat input and 2209 filler rod. The yield strength, ultimate tensile strength, and elongation of the FSWed joint are enhanced over the GTAW weldment by 21%, 41%, and 66% and over the BM by 65%, 33%, and 54%, respectively. EBSD investigation showed a significant grain refining after FSW with grain size average of 1.88 µm for austenite and 2.2 µm for ferrite.

## 1. Introduction

Compared to ferritic and austenitic stainless steels, duplex stainless steel (DSS) has excellent strength and corrosion resistance in heavy-duty engineering applications due to a dual-phase structure (nearly 50% ferritic (α) phase and 50% austenitic (γ) phase) [1,2,3,4,5]. This unique microstructure promotes DSS advantages over each austenitic and ferritic stainless steel. It was reported that [4] stress corrosion cracking and the elastic limit of austenitic stainless steel improves with the presence of the α-phase. Moreover, the presence of the γ-phase leads to toughness enhancement of the duplex stainless steels by hindering grain growth of the α-phase [5]. Thus, DSS is recommended extensively for use in many applications, such as chemical and petrochemical industries, marine industry, water desalination pipes, and liquefied natural gas pipes, besides being used in many mining applications [3,5]. 

Consequently, DSS is considered a proper choice in many engineering applications instead of ferritic and austenitic stainless steels [6]. Among duplex stainless steels, 2205 DSS is regarded as the most common type of this class of steels. This grade is widely used in oil and gas industries, seawater components, and power generation applications [4,6]. The joint efficiency of the 2205 DSS weldments is mainly structure-sensitive and depends on the type of welding method. The 2205 DSS can be welded using high arc energy fusion welding methods such as gas metal arc welding (GMAW) [7], gas tungsten arc welding (GTAW) [8,9], and shielded metal arc welding (SMAW) [2]. The GTAW process promotes efficient and clean weldment compared with other fusion welding processes [10]. It was reported that the weld zone (WZ) of GTAW welds may show an acceptable γ/α ratio by using a special filler rod. However, the weld microstructure features are not similar to its base metal microstructure [11], and the productivity is low, as the maximum thickness that can be welded in single pass cannot exceed 3 mm [2,12]. The GTAW process using nickel-enriched filler rod ER 2209 provides an acceptable α/γ ratio in the weldments [13]. It was reported that the chemical composition of the filler rod plays a significant role in the γ-phase reformation than the applied cooling rate [14]. 

FSW, as a solid-state joining technique, has become increasingly prominent in the joining and welding of duplex stainless steels (DSS) [15]. In 2205 DSS, optimum properties are obtained when the material has equal proportions of austenite and ferrite [16]. This desired phase ratio changed during fusion welding processes and promoted more ferrite formation in the weld zone as a result of remelting and solidification of materials [17]. In addition, it is recommended to weld DSSs within the range of 0.2–1.5 kJ/mm to avoid precipitation of brittle intermetallic phases [18]. On the other hand, solid-state welding techniques such as FSW seem to be a suitable technique in this regard [19]. This process minimizes the ferritization problem of DSSs during thermal welding cycles due to their solid-state nature and reduces the common problems associated with fusion welding. During the FSW process, the microstructure of the base material experiences both high temperature and severe deformation caused by the stirring tool. The coexistence of these two factors activates the occurrence of some softening mechanisms throughout the material, which results in a kind of fine microstructure commonly found during the hot deformation processes [20]. 

Friction stir welding (FSW) of soft materials such as aluminum and magnesium alloys became a common activity of welding due to the ability of the tool steel material to weld such materials at minimum cost and risk of failure [21,22]. However, the risk of failure and cost are still very high in case of tool materials used for FSW of hard metals, such as steel [23], titanium and nickel alloys [24]. This is mainly due to the high temperature and severe stress experienced during FSW [25]. Tool materials for FSW of high softening temperature alloys must exhibit excellent properties at temperatures in excess of 900 °C [25]. There are various types of materials used in FSW of high softening temperature materials, such as WC-based materials [26], W–Re [27], and PCBN materials [28]. However, the FSW tool is subjected to severe stress and high temperatures, particularly for the welding of hard alloys such as steels and titanium alloys and the commercial application of FSW to these alloys is now limited by the high cost and short life of FSW tools [25]. However, due to high temperatures and pressures required in the manufacturing of PCBN, the tool costs are very high [25]. Owing to its low fracture toughness, PCBN also has a tendency to fail during the initial plunge stage [25]. Thus, the WC material represents a cost-effective option for FSW of hard metals [26] and hard composites [29,30] if the tool life can be extended. An important parameter that can be considered to extend tool life is the geometry of the tool pin and the shoulder [31]. In addition, reducing the forces experienced by the tool, either upon plunging and or during FSW, can improve the WC tool life. In this respect, having grooves along the joint line will significantly reduce these forces. In addition, the idea of groove filling using FSW is almost new and innovative, in respect to filling without using a filler rod. The advantage of FSW over other welding techniques, such as laser beam welding [32] as a welding technique without the need for filler rods, is the lower heat input. Thus, this work aims to introduce FSW as a groove filling joining technique to weld DSS in petroleum services companies instead of the GTAW fusion technique. 

## 2. Materials and Methods

### 2.1. Material

SAF 2205 DSS plates of 6.5 mm in thickness were used as the base material. The workpieces for welding were cut from the as-received plates for both GTAW and FSW processes, 200 mm in length and 100 mm in width. The chemical composition of the base material DSS is listed in Table 1. The filler rod used in the GTAW process is AWS A5.9 ER2209, which contains 3.68% more nickel than 2205 DSS. The increased nickel content in the filler rod stabilizes the austenite phase in the weldments. The chemical composition of the filler rod is given in Table 2. The chemical compositions of the base material and the filler rod are obtained from the datasheet provided by the supplier. 

### 2.2. Welding Procedure

**Figure 1:**
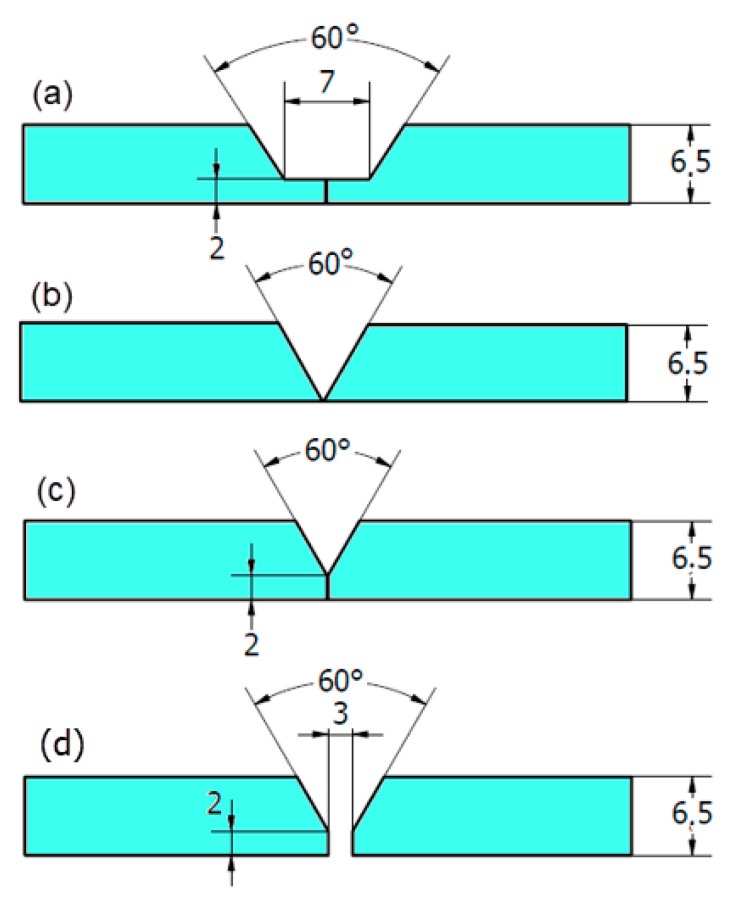
Groove joint designs (**a**–**c**) for the FSW process and (**d**) for the GTAW process [33].

For the FSW, three different groove geometries without root gap were designed and machined, as shown in Figure 1: (a) a 60° U-shaped groove with a 2 mm root face, (b) a 60° V-shape groove without a root face, (c) a 60° V-shape groove with a 2 mm root face, and (d) for the GTAW 60° V-shape groove with a root face and root gap. Before welding, the samples were mechanically cleaned using a stainless-steel wire brush to remove surface oxides and contaminations. 

**Figure 2:**
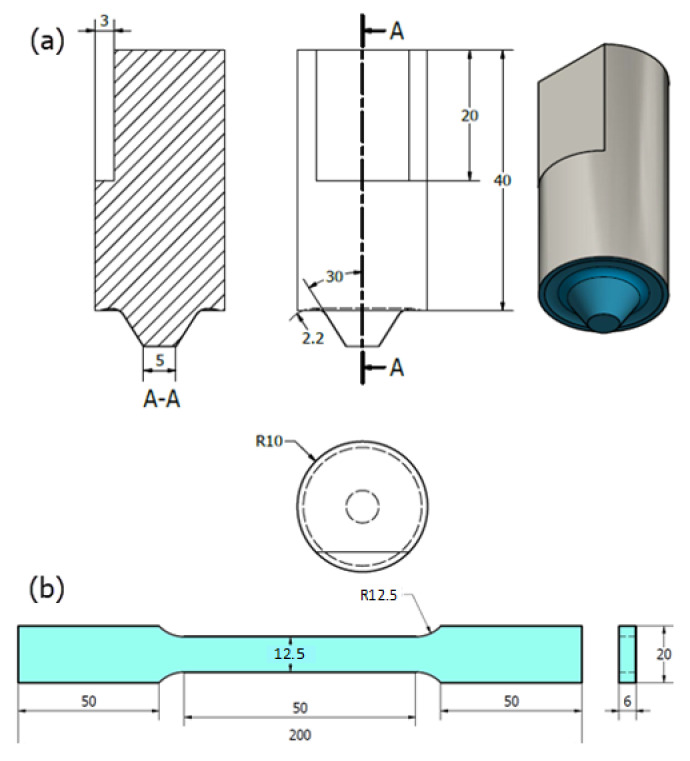
Schematic of (**a**) drawings of the WC FSW tool and (**b**) the tensile test sample (dimensions in mm).

The FSW tool was designed with a tapered pin geometry. The shoulder diameters, pin tapered angle, pin tip, and pin length were selected as 20 mm, 30°, 5 mm, and 5.5 mm, respectively. Due to the high frictional stress and heating during FSW of DSS; tool, shoulder, and pin were fabricated from tungsten carbide (WC) [1]. Figure 2a shows the drawings of the WC tool. 

Based on preliminary FSW experimental trials and also the available data in the literature [34], the applied optimum FSW welding parameters were a rotational speed of 300 rpm, a travel speed of 25 mm/min, and a tilt angle of 3°. The FSW experiments were conducted using the FSW machine model (EG-FSW-M1) [35]. For comparison between FSW and the fusion welding technique, gas tungsten arc welding (GTAW) was chosen and applied to weld DSS 2205 in the butt joint. Among fusion welding techniques, GTAW is used extensively to weld duplex materials in the petroleum and petrochemical industries. The used geometry joint design for the GTAW process was a 60° V-shape groove angle with a 2 mm root face and root gap of 2 mm; Figure 1d. The joint was prepared according to a joint design within the approved welding procedure specifications.

The GTAW process was performed using the DCEN-GTAW machine with tungsten electrode 2.4 mm type EWTh-2, arc length 2.4 mm, and argon flow rate 15 l/min. The GTAW process was carried out using the prequalified welding procedure specifications (PQWPS) with welding parameters, as shown in Table 3. 

### 2.3. Joints Evaluation and Characteriztion

Radiographic inspection test (RT) was carried out according to ASME V and ASME VIII on the welded joints using a Gamma-ray camera (Model 880 MAN-027, NSW, Australia) with an Iridium-192 source using D7 radiographic films. Microstructural analysis of the welds was performed using optical microscope (Olympus model: BX41M-LED, Tokyo, Japan). The transverse cross-sections were prepared through basic metallographic steps according to ASTM E-3 and ASTM E-2014 up to 2400 grit and then mechanically polished using alumina suspension of 0.05 μm. This was followed by cleaning with acetone, and drying and electrochemical etching using 20 g KOH in 100 ml H_2_O solution. The phase composition in the stir and fusion zones of the FSWed and GTAWed butt joints were examined by X-ray diffraction (XRD, Siemens Incorporating, Munich, Germany) analysis using Siemens-D5000 with Cu Kα radiation (wavelength λ = 0.15406 nm) at 40 kV and 30 mA in the 2θ-range 20–110° and step size of 0.03^o^. The ferrite number of base material (BM), heat affected zone (HAZ), and weld zone (WZ) was evaluated for the joints welded by FSW and GTAW using a ferrite–scope (FERITSCOPE MP30—Fischer Company, Worcestershire, UK). 

The Vickers hardness tester model (Qness model Q10 M, Golling, Austria) was used to obtain the hardness values across the transverse cross-section at a spacing of 1 mm with a load of 98 N and 15 s holding time. In order to evaluate the welding joint efficiency, tensile test specimens were cut perpendicular to the welding direction according to ASTM E8. Figure 2b shows the standard tensile test sample dimensions that were used as a reference in the preparation of the joints tensile samples; the tensile test sample thickness was varied according to the thickness of the weld zone i.e., the thickness at the weld zone was used as the tensile sample thickness in each case, which means that the extra thickness material above that has been machined. The test was carried out at room temperature at a ram head-speed of 0.5 mm/min using a universal test machine (Instron 4208, 300 kN capacity, Norwood, MA, USA).

## 3. Results and Discussions

### 3.1. Mechanism of FSW with Groove Filling

**Figure 3:**
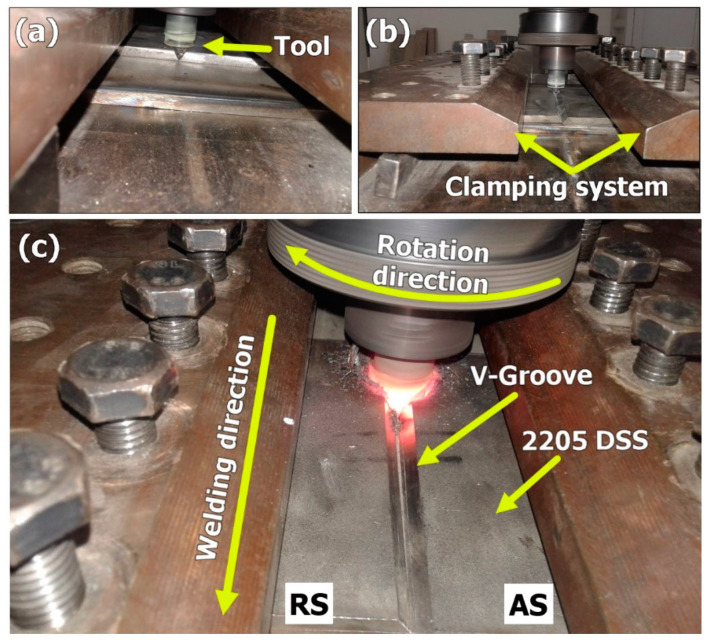
Photographs taken during FSW of 2205 DSS with groove filling. (**a**) Front view during FSW tool plunge, (**b**) back view during FSW tool plunge and (**c**) front view during FSW.

**Figure 4:**
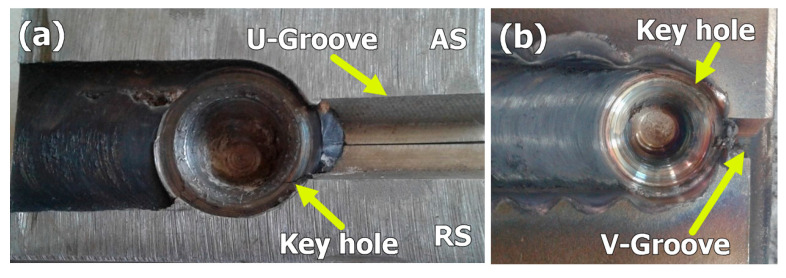
Key hole after FSW of 2205 DSS (**a**) U shape groove with root. (**b**) V shape groove without root.

**Figure 5:**
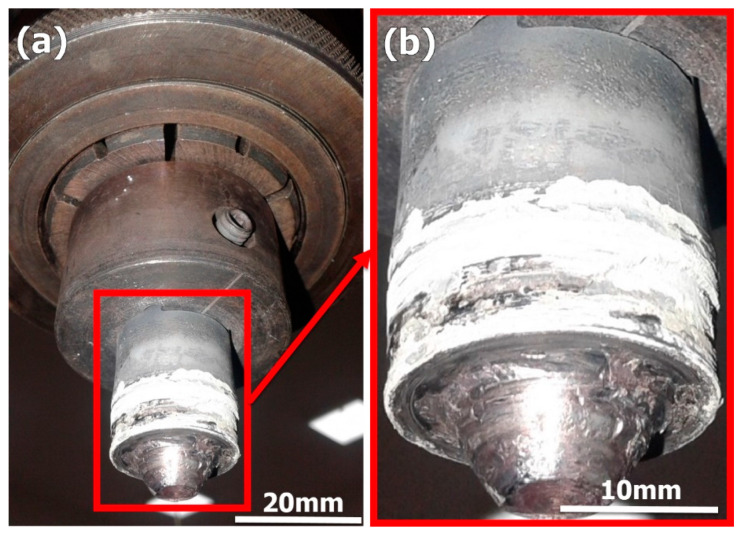
(**a**) WC FSW tool with the holder arrangement after eight trials of duplex stainless steel welding with the WC tool enlarged in (**b**).

It has been known since the invention of the FSW process that this solid state welding process cannot be used for welding with the need for filling of grooves. In this work, a feasibility study for the use of FSW in the welding of butt joints with grooves was carried out. Figure 3 shows the FSW tool plunging into the groove from the front in (a) and from the back in (b). It can be noted that the existence of the groove facilitates the plunging stage and is expected to reduce tool wear and extend tool life. After complete plunging, as shown in Figure 3c, the material starts to flow around the tool and a pole is formed around the tool that becomes stable with only little flow in front of the tool that does not affect the joint behind the tool. It can be observed that this pole becomes stable with the travers of the tool as can be seen from the existing hole pictures shown in Figure 4 for a U-shaped groove with root (a), and a V-shaped groove without root (b). This joint configuration is expected to facilitate tool plunging, reduce tool resistance forces, and reduce the heat input. This will directly enhance the tool life and joint properties. Figure 5a shows the WC FSW tool with the holder arrangement after eight trials of 2205 DSS welding with some trace of material sticking on the WC tool. In addition, the enlarged image of the WC tool in Figure 5b shows the stability of the tool features, which indicate that no wear took place under the condition of having the grooves and only oxidation can be noted on the shoulder outer surface due to the high thermal cycles experienced. The stability of the WC after repeated plunging and welding for eight times can be attributed to the existence of the groove, which reduces the tool resistance forces. 

### 3.2. Visual and Radiographic Inspection

**Figure 6:**
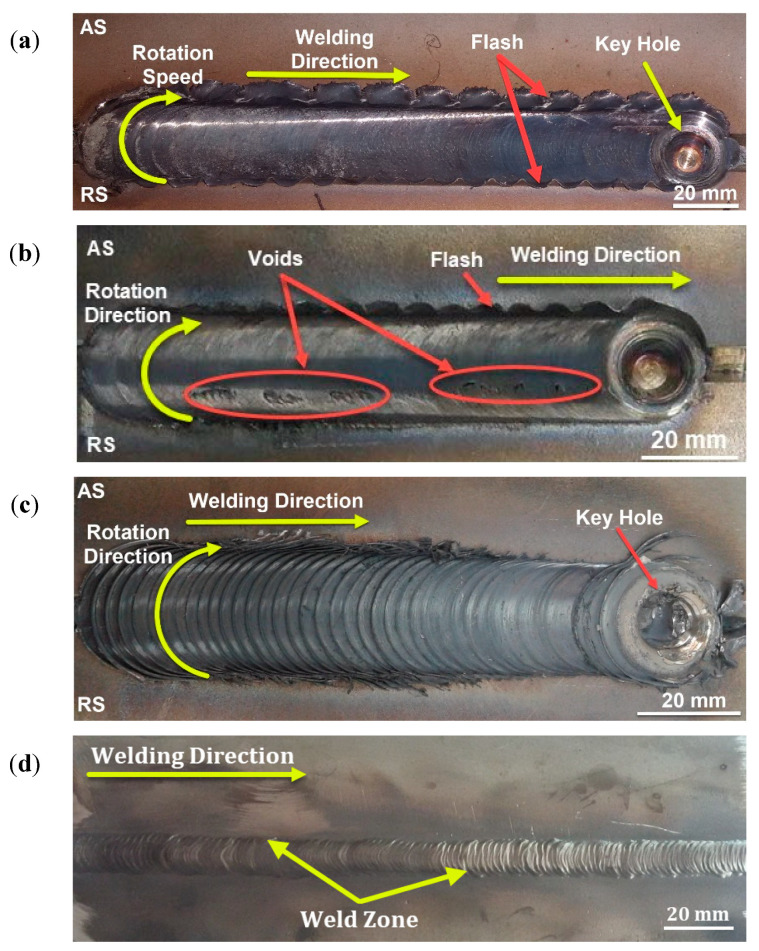
Top view of FSW welds (**a**) FSW, U-shaped groove with root; (**b**) FSW, V-shaped groove without root; (**c**) FSW, V-shaped groove with root face; and (**d**) GTAW, V-shape groove with root face and gap GTAW weld for 2205 DSS butt joints [33].

**Figure 7:**
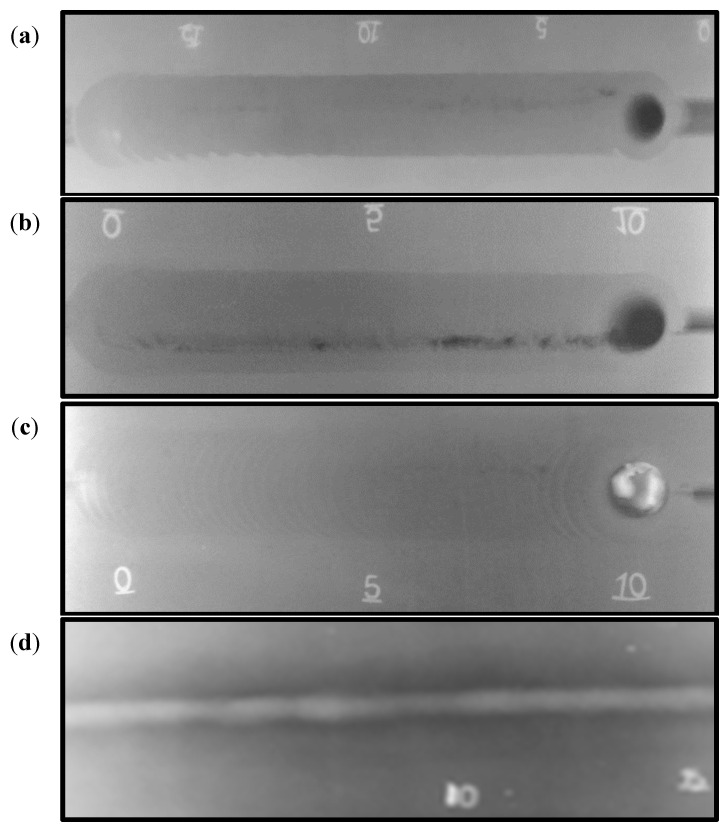
Radiographic films of FSW welds: (**a**) FSW, U-shaped groove with root; (**b**) FSW, V-shaped groove without root; (**c**) FSW, V-shaped groove with root; and (**d**) GTAW, V-shaped groove with root face and root gap GTAW weld for 2205 DSS [33].

Figure 6 shows the top view of the FSWed 2205 DSS butt joints produced with self-filling of different groove designs. It can be observed that in all cases, the grooves filled successfully, with some defects noted in the U- and V-shaped grooves without a root face mainly due to the large volume of the grooves that require large volumes of material to be soundly filled. For example, the U-shaped joint produces a good surface appearance with extensive flash. Moreover, around 2 mm reduction in weld pass thickness was detected, compared to the base material, to achieve filling of the groove; Figure 6a. This loss in thickness is due to the lack of material flow and the large volume of the joint groove design (Figure 1a). The produced weldment of the V-shaped groove design without root reveals a partially good appearance with less flash. Some surface voids along the joint welding length at the retreating side are seen; Figure 6b. Moreover, around 1 mm reduction in weld pass thickness was measured, compared to the DSS BM. These top surface features are also related to the lack of material filling and flow as a result of the large volume of the groove-shaped design (Figure 1b). The obtained joint using the V-shape with root face design showed a good surface appearance, with little flash and minimum reduction in thickness. For comparison purposes, the same lap joint of 2205 DSS was produced using the GTAW method with a V-shaped groove design with root and 3 mm root gap (Figure 1d). Figure 6d shows the top view of the GTAW butt weld of 2205 DSS. It can be considered to be of acceptable appearance without surface defects. The radiographic inspection reveals internal defects for the butt joints produced using U-shape and V-shape without root face designs, as shown in Figure 7a,b, respectively. Furthermore, nearly sound 2205 DSS butt joints were obtained for both FSW butt joints using a V-shaped groove design with root and GTAW butt joint, as given in Figure 7c,d, respectively. 

### 3.3. Macrostructure and Microstructure

**Figure 8:**
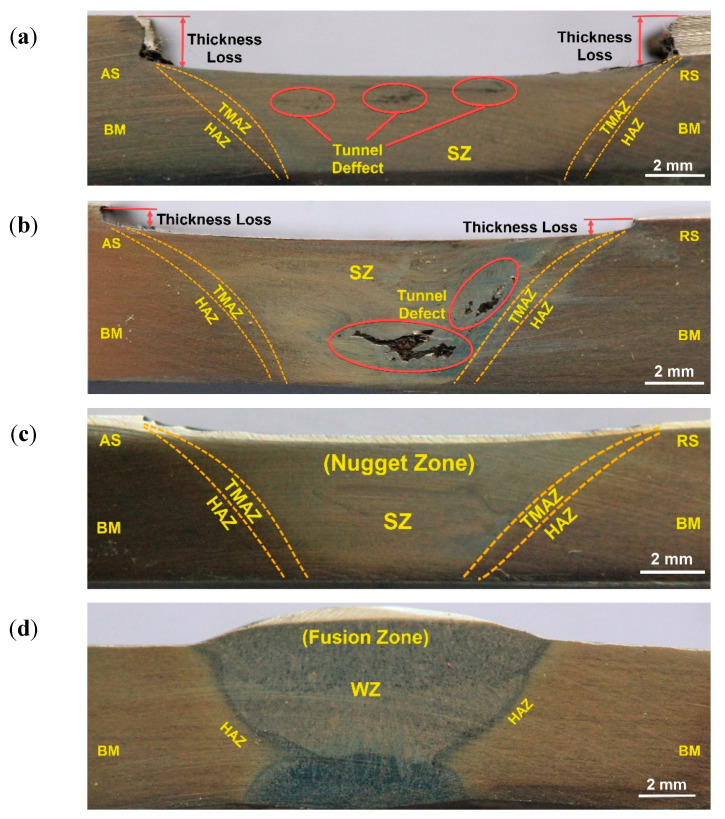
Macrostructure of FSW welds: (**a**) FSW, U-shaped groove with root; (**b**) FSW, V-shaped groove without root; (**c**) FSW, V-shaped groove with root face; and (**d**) GTAW, V-shaped groove with root face and root gap, GTAW weld for 2205 DSS butt joints.

Figure 8a–c shows the transverse cross-section macrographs of the FSWed butt joints produced using different groove designs. Figure 8d represents the cross-section macrograph of the GTAWed butt joint. For the FSWed joints, three distinguished zones—stir zone (SZ), thermomechanically affected zone (TMAZ), and heat-affected zone (HAZ)—are observed [35]. Furthermore, the interface between the SZ and the TMAZ is very distinguished on the advancing side, while it is more scattered on the retreating side, which is due to the alignment of the rotation direction, transverse direction at this side, and also the more refined grains at the advancing side than that at the retreating side [36]. There are tunnel defects and 2 mm thickness loss of the 2205 DSS butt joint using U-shaped groove design without root, as shown in Figure 8a. These defects are due to the large groove volume of the joint. In addition, there are internal defects observed (Figure 8b) for the butt joint welded using the V-shaped groove without root; these defects are tunnel defects. Moreover, the reduction in thickness for this joint is around half reduction thickness for the joint welded using the U-shaped groove design with root. These defects may also be ascribed to improper groove joint design. For the welded joint using a V-shaped groove with root, it was noted that the cross-section is defect-free and a very slight reduction in thickness was detected. Based on visual inspection, radiographic examination, and macrograph investigation, it can be concluded that a 60° V-shaped groove with 2 mm root face without root gap is a proper joint design for the FSW filling groove process to produce a sound joint. In comparison, a defect-free joint was obtained with a V-shaped groove with 2 mm root face and 3 mm root gap produced using GTAW, as shown Figure 8d. Moreover, the two distinguished zones, WZ and HAZ, can be detected as fusion welding features [37].

**Figure 9:**
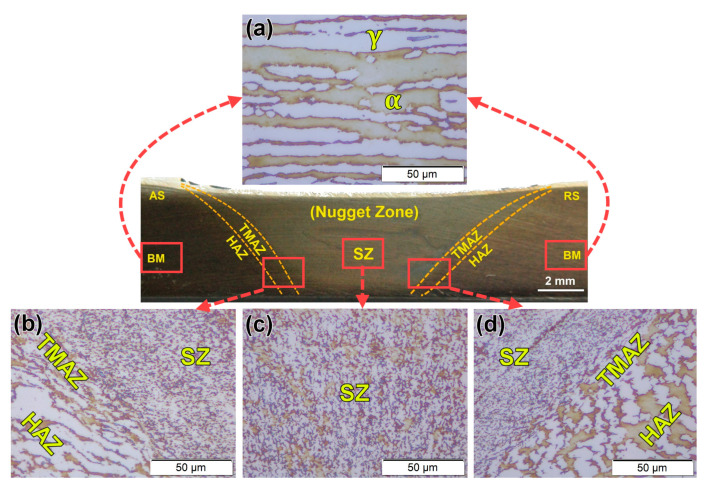
Optical microstructure of the FSWed 2205 DSS butt joint using the V-shaped groove with root face: (**a**) BM, (**b**) SZ/TMAZ/HAZ on the advanced side, (**c**) SZ, and (**d**) SZ/TMAZ/HAZ on the retreating side.

**Figure 10:**
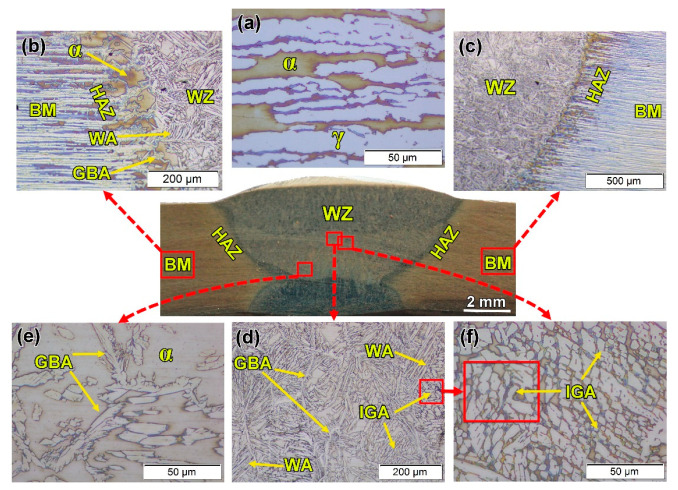
Optical microstructure of the GTAWed 2205 DSS butt joint using the V-shaped groove with root face and root gap: (**a**) BM, (**b**,**c**) WZ/HAZ/BM at high and low magnifications, respectively, (**d**–**f**) WZ.

Figure 9 shows the microstructural features at the different zones of the sound FSWed joint produced using the V groove, 60° groove angle, 2 mm root face joint design, (a) base metal (b) TMAZ in the advancing side (AS), (c) SZ, and (d) TMAZ in retreating side (RS). A typical lamellar microstructure of 2205 DSS consists of γ-phase islands in the α-phase matrix. These γ islands have a pronounced orientation in the α matrix, parallel to the rolling direction, as given in Figure 9a. This lamellar structure of the α and γ phases of DSS BM is recrystallized and grain refined to give the equiaxed grains (Figure 9c) due to the severe plastic deformation enforced by the FSW tool and the high frictional heating. It has been reported that SZ is the core of the FSWed area, where the FSW tool causes major material agitation and heating. Consequently, temperature and deformation are at their extreme values in the SZ compared to TMAZ [38]. At a little distance away from the centerline of the 2205 DSS FSW pass, TMAZ is noticeable (Figure 9b,d). It is characterized by lower temperature and deformation, compared to the SZ [39,40]. The refined equiaxed grains in the SZ and the formation of elongated grains in the TMAZ promotes a sharp interface between the two zones (Figure 9b,d). After TMAZ moves toward the BM, HAZ appears, as shown in Figure 9b,d). It is far away from the tool-stirring zone (SZ). Thus, it only suffers from a cycle of heating and cooling without any deformation. Consequently, the microstructure features of this zone are close to BM with a slight grain growth in some FSWed joints [34]. The DSS solidification during the fusion welding is a type of ferrite prior. This means that from the liquid state, the grains of α-phase nucleate and grow preferentially, and different morphologies of γ grains are subsequently formed [41]. Furthermore, the thermal cycle across the DSS welds is highly heterogeneous and complex, especially for the multi-pass welding as in the GTAW process [42]. Figure 10 shows the optical microstructure of the cross-section of the GTAWed joint—the BM (Figure 10a) and the different welding zones: HAZ (Figure 10b,c), and WZ (Figure 10d–f). High and low magnifications of the HAZ are illustrated in Figure 10b,c, respectively. It can be seen that a relatively wide HAZ width was obtained. The HAZ coarse-grained structure zone (Figure 10b) adjacent to the fusion zone line could be stemmed from nearly complete γ-phase dissolution on the heating cycle and subsequent α-phase grain growth. The HAZ microstructure consists of large α grains with continuous networks of γ-phase at the α-grain boundaries (GBA) and tiny ones of Widmanstatten austenite (WA) (Figure 10). However, the heat input at the WZ is higher than that of the HAZ; therefore, different γ grain morphologies are likely to be found, including GBA, the intragranular γ-precipitates (IGA), and WA. Eghlimi et al. [43] reported that the GBA forms at a temperature range of 800–1350 °C. The WA grains precipitated from the grain boundary γ-phase, as shown in the WZ (Figure 10b,d). The WA side laths, nucleating from grain boundary γ-phase, form along grain morphologies orientations in the α matrix. Ramirez et al. [44] reported that the WA forms at a temperature range of 650—800 ^o^C. The IGA nucleates within α grains (Figure 10d,e). It also maintains a crystallographic orientation with the α-phase. The IGA grains are finer compared with the GBA and WA grains. These microstructure features are mainly considered the microstructures of 2205 DSS welded by fusion welding techniques, as reported in many previous works [2]. Figure 9 is simple and consists of fine recrystallized ferrite and austenite. The microstructure after GTAW, shown in Figure 10, is quite complex and consists of different austenite morphologies such as GBA, IGA, and coarse ferrite grains with a continuous network of austenite.

### 3.4. XRD Assessment

**Figure 11:**
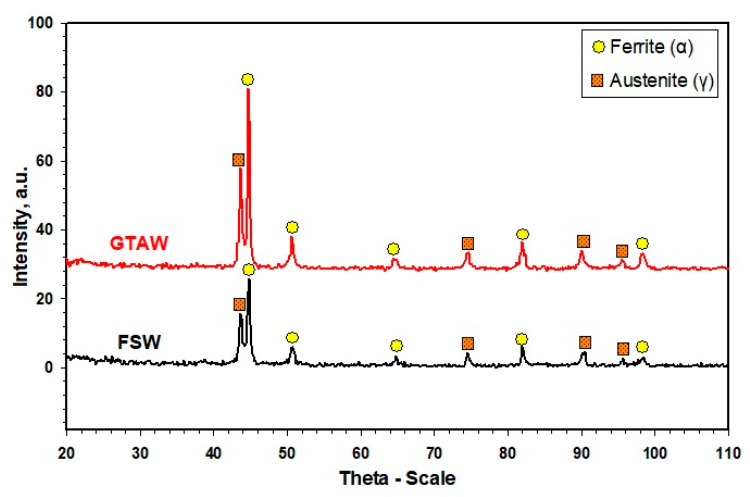
XRD patterns of the 2205 DSS FSWed joint produced using a 60^o^ V groove with root face and GTAWed butt joints.

Figure 11 shows the X-ray diffraction patterns of the FSWed and GTAWed butt joints of the 2205 DSS. Only peaks of α and γ phases can be seen. It seems that sigma phase (σ) was not found in the WZ for both joints or its amount may be lower than 5%, which is well-known to be the detection average limit by XRD. It should be mentioned that σ was also not observed in microstructures’ investigation. It was reported that [13,45] the weld thermal cycle of DSS has a crucial influence on the σ-phase precipitation. The σ-phase is normally formed after long holding times at temperatures from 650 to 950 °C.

### 3.5. Measured Austenite Content 

**Figure 12:**
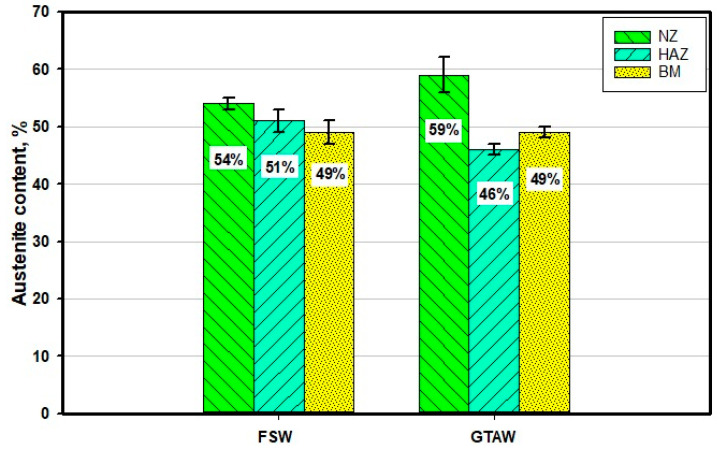
Austenite contents (%) in microstructural zones of the FSWed joint produced using a 60^o^ V groove with root face and GTAWed 2205 DSS obtained using a ferrite-scope type of FERITSCOPE MP30. Note: for the FSWed sample, WZ represents the SZ and HAZ represents both TMAZ/HAZ.

Figure 12 shows the γ content measured using the ferrite–scope type of FERITSCOPE MP30 across the 2205 DSS welded joints produced by GTAW and FSW techniques. The γ-phase contents in BM, HAZ, and WZ of the 2205 DSS GTAW welded joint are 49%, 46%, and 59%, respectively, as given in Figure 12. It can be noted that the γ-phase percentage in the WZ of the 2205 DSS joint is 59%, which is higher than the BM. This increase in γ-phase can be ascribed to the use of filler rod ER2209 during the 2205 DSS GTAW method, containing higher Ni than the 2205 DDS BM [46]. Moreover, the γ content in HAZ is 46%, which is slightly less than that of BM. This decrease in γ-phase percentage can be attributed to the heating/cooling cycle during the GTAW process [46]. The γ-phase content of the SZ in the 2205 DSS welded joints using FSW is shown in Figure 12. The γ-phase contents are 54% across the SZ and 51% across the HAZ/TMAZ zone, which is slightly higher than that of the BM. The increase in the γ-phase content in the SZ is ascribed to the exposure to the high temperature during the FSW process at the applied welding conditions of 300 rpm and 25 mm/min. This high-temperature exposure has resulted in the transformation of a portion of the α-phase into γ-phase upon cooling [47]. It can be said that the α/γ ratios in the weld zone are more suitable for joints welded by FSW than by GTAW.

### 3.6. EBSD Investigation

**Figure 13:**
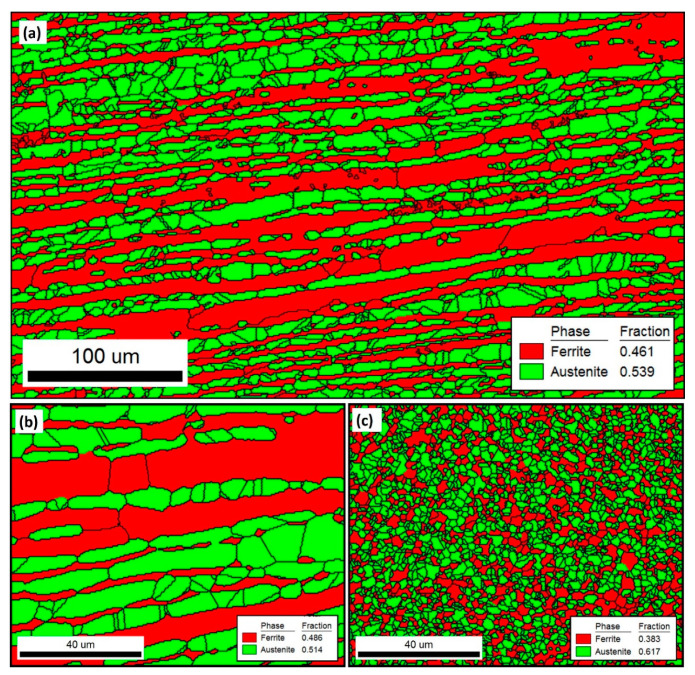
Phase-colored maps (austenite in green and ferrite in red) for the base material (**a**,**b**) and the FSWed joint stir zone (**c**) with the high angle boundaries (HABs) >15^o^ in black lines superimposed. The data in (**a**) is obtained using a 1 µm step size while the data in (**b**,**c**) are obtained using a 0.5 µm step size.

**Figure 14:**
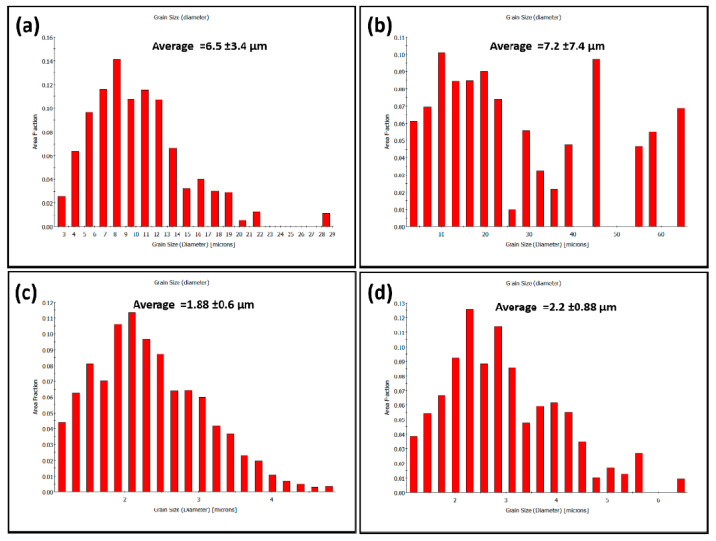
Grain size distribution of BM (**a**) austenite, (**b**) ferrite and NG of FSWed joint (**c**) austenite, (**d**) ferrite.

Figure 13 shows the phase-colored maps (austenite in green and ferrite in red) for the base material (a and b) and the stir zone of the FSWed joint produced using a 60^o^ V groove with root face (c) with high angle boundaries (HABs) >15^o^ in black lines superimposed. It can be observed that the base material microstructure consists of highly elongated grains of both ferrite matrix (red) and austenite islands (green) with volume fractions of 0.46 and 0.54, respectively, as indicated on the map. It should be noted here that the volume fraction is slightly changed after changing the data acquisition step size as after using 0.5 µm step in (Figure 13b), it becomes 0.49 and 0.51, respectively, which almost reaches the ideal phase fraction of 1:1. The elongated ferrite grains are more continuous while the elongated austenite grains are divided into more fine grains with HABs. Figure 14 shows the grain size distribution of the base material austenite (a) and ferrite (b). It can be observed that the austenite has almost random distribution and grain size ranging from 3 µm up to 22 µm with an average of 6.5 µm. While the ferrite has non-random distribution and wider grain size range from 3 µm up to 60 µm with an average of 7.2 µm. Figure 13c shows the phase-colored map for the NG zone after FSW. Clearly the grain morphology in the NG zone is completely different than that observed in the base material. Significant grain refining can be observed with equiaxed grain morphology for both ferrite and austenite. The stir zone in the FSW experiences very high degree of plastic strain at high temperatures both generated by the FSW tool stirring of the material surrounding the tool [19,48]. This condition has resulted in a dynamic recrystallization process to take place in the stir zone and the formation of the equiaxed fine grain structure. The grain size distribution after FSW is shown in Figure 14c,d for austenite and ferrite phases, respectively. Both phases show grain size random distribution with range between 1.2 µm to 4.7 µm for austenite with an average of 1.88 µm and 1.2 to 6 µm for the ferrite phase with an average of 2.2 µm. The volume fraction of the two phases are 0.62 and 0.38 for austenite and ferrite, respectively. It can be noted that there is an increase in the austenite percentage in the stir zone, which is consistent with the obtained result using the ferrite–scope device with slight higher percentage obtained using EBSD. This might need a smaller step size to reduce the percentage austenite, as observed when two different step sizes are used in the case of BM. The results obtained in terms of grain refining and the percentage of phases are in agreement with the results reported in the literature [49].

**Figure 15:**
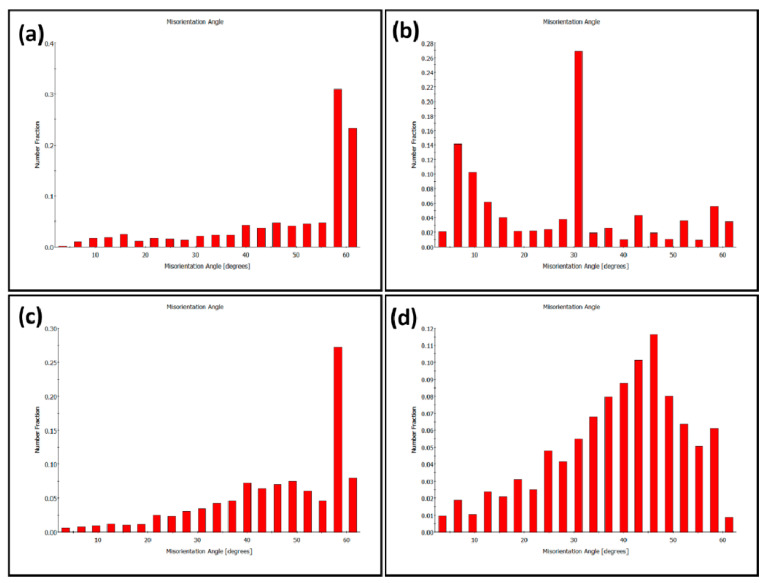
Misorientation angle distribution of BM (**a**) austenite, (**b**) ferrite and NG of FSWed joint (**c**) austenite, (**d**) ferrite.

From the misorientation angle distribution shown in Figure 15, it can be noted that the BM is dominated by HABs with non-random distribution for both phases, austenite (a) and ferrite (b). A high density of twin boundaries at 60° can be observed in the austenite, as it can also be observed in the GB maps superimposed in Figure 13. After FSW, both phases (c) austenite and (d) ferrite misorientation angle distributions show random distribution, which is the typical distribution of the recrystallized material. The density of twin boundaries in the austenite can still be observed at the same misorientation angle of around 60°.

**Figure 16:**
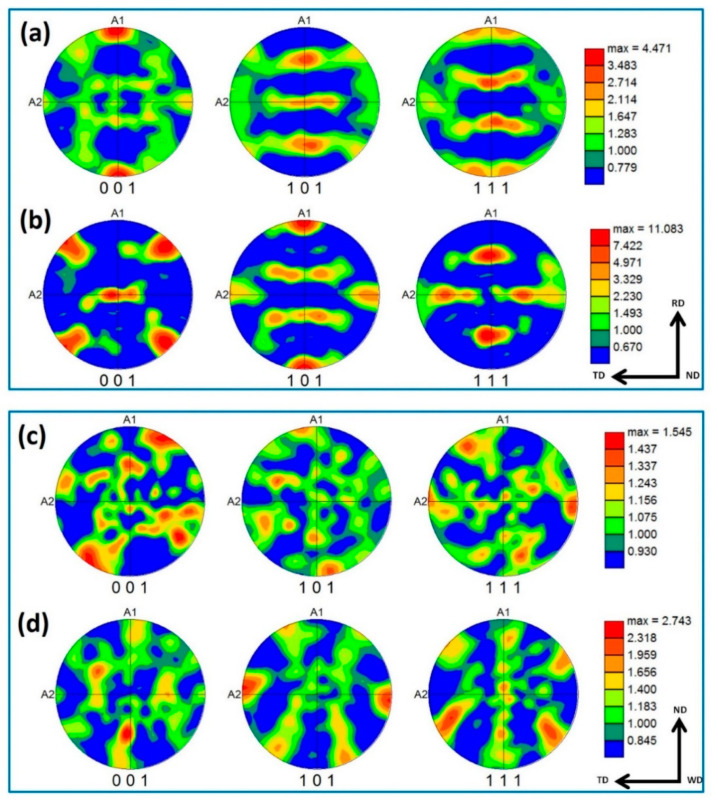
001, 101, and 111 pole figures of BM (**a**) austenite, (**b**) ferrite and NG of FSWed joint (**c**) austenite, (**d**) ferrite.

Crystallographic texture before and after FSW is presented in Figure 16 as 001, 101, and 111 pole figures (a,b for BM austenite and ferrite, respectively), (c,d for FSWed NG zone austenite and ferrite, respectively). Both austenite and ferrite of BM show the typical fcc and bcc rolling texture with more strong texture (~ 11 times random) in case of the ferrite than that of the austenite (~ 4 times random). After FSW, the texture for both phases is weak, with only about two times random, resembling the shear texture of both phases. 

### 3.7. Hardness Distribution 

**Figure 17:**
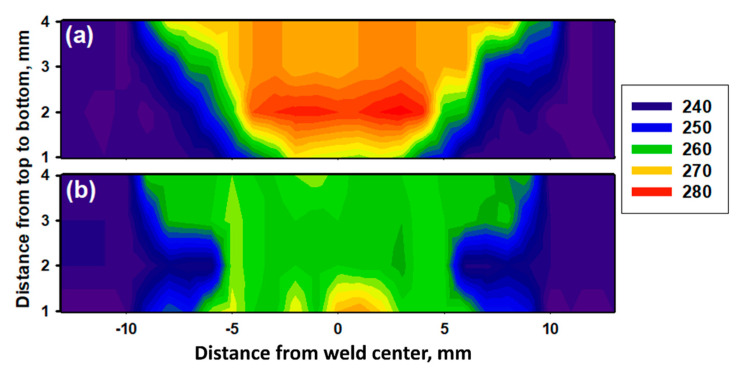
Vickers hardness maps of the DSS 2205 welds (**a**) FSWed joint produced using a 60o V groove with root face and (**b**) GTAWed joint.

Figure 17 exhibits the variation of hardness across the 2205 DSS butt joints welded by (a) FSW and (b) GTAW. In general, it can be seen that the hardness values in the WZ of FSWed joint is higher than that of the joint welded by the GTAW process. For the FSWed joint, it can be seen from Figure 17a that the hardness reaches a maximum value of 280 HV at the SZ and gradually decreases by passing from TMAZ and HAZ to be 260 HV and 250 HV, respectively. The severe plastic deformation accompanied by the maximum temperature in the SZ during the FSW process leads to the most effective microstructure modification in terms of grain refining (Figure 9c) due to dynamic recrystallization [35,50], which justifies the highest hardness value in this region. In the TMAZ, lower plastic deformation and temperature are introduced during the FSW, leading to less grain refinement and lower hardness values. In HAZ, deformation is absent, and the DSS material only undergoes a thermal cycle [51]. This validates the lowest hardness values close to that of the BM in this region. For the GTAWed joint, it can be seen from Figure 17b that the hardness reaches a maximum value of 265 HV at the WZ and gradually decreases by passing from HAZ and BM to be 250 HV and 240 HV, respectively. The fluctuation in hardness values can be ascribed to the microstructural features observed [13]. The existence of more intergranular γ (Figure 10d) enhances the hardness of WZ [52], while the presence of much coarser α grains decreases the hardness of HAZ. The same trends were obtained in other works [46].

### 3.8. Tensile Properties

**Figure 18:**
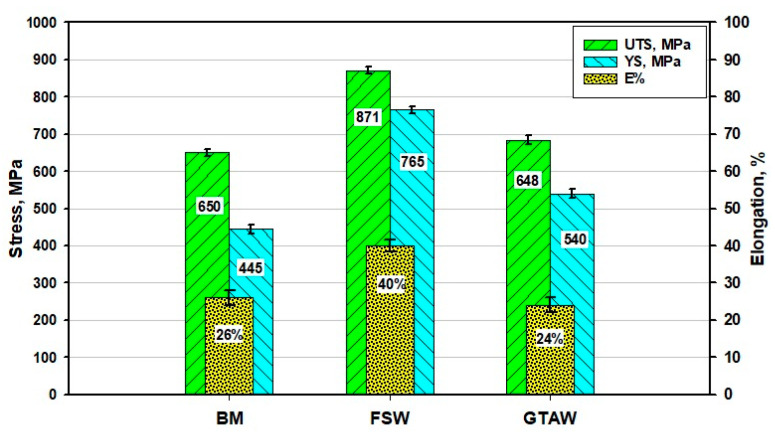
Tensile properties of DSS 2205 base material and FSWed joint produced using a 60^o^ V groove with root face and a GTAWed joint.

Figure 18 shows the tensile properties in terms of yield stress (Ys), ultimate tensile stress (UTS), and elongation (E%) of the welded joints by FSW and GTAW processes compared to the BM. It is obvious that the FSWed joints have much higher tensile properties (Ys, UTS, and E%) than that given by both GTAWed joints and the BM. This is in agreement with the measurements of hardness shown in Figure 16. It can be seen in Figure 17 that the Ys, UTS, and E% of FSWed joints are enhanced over the BM by 65%, 33%, and 54%, and over the GTAW weldments by 21%, 41%, and 66%, respectively. This enhancement of the tensile properties is mainly ascribed to the main features of grain modification during the FSW process [53]. Ghadar et al. [38] reported significant improvement of ultimate tensile strength and fracture strain for friction stir processed 3.5 mm 2205 DSS and ascribed this enhancement in tensile properties to the grain refining. It can be said that the grain size in the WZ is a dominant factor governing mechanical properties. Furthermore, only around 16% improvement in the Ys is detected for the GTAWed joints over the Ys of the BM with no remarkable increase in the UTS. This enhancement in yield stress may also be ascribed to the grain refining in the WZ [42]. 

## 4. Conclusions

The effect of groove joint design on the microstructure and mechanical properties of 6.5 mm plates butt joints 2205 DSS welded by FSW and GTAW techniques were examined and evaluated. The following conclusions can be drawn:

1. The FSW used to weld 2205 DSS of 6.5 mm thick with groove filling is shown to be feasible.
2. The optimum groove design to produce a sound joint with groove filling is the V-shaped with 60^o^ groove angle and 2 mm root face.
3. The use of the groove in the butt joint of DSS 2205 extended WC tool life due to the reduction in tool resistance forces during plunging and welding.
4. The heat input and 2209 filler rod used in the GTAW process produce different γ-phase morphologies, such as GBA, IGA, and WA, besides the α phase in the WZ. In comparison, the friction stir weld has only two-phase microstructures: α and γ with significant grain refining.
5. The α/γ ratio in the weld zone is influenced by welding techniques (solid-state welding and fusion welding) and their parameters in terms of welding temperature, joint groove design, and the presence or absence of filler rod. Compared with the BM that attains an α/γ ratio of 51/49, GTAW promotes a ratio of 41/59 while FSW produces a ratio of 44/56.
6. Grain refining in the NG zone of the FSWed joint has been quantified to be 1.88 µm for austenite and 2.2 µm for ferrite from about 6.5 µm and 7.2 µm in the BM phases, respectively.
7. Significant hardness improvements have been detected for the weld joint produced by FSW than that produced by GTAW.
8. The FSWed 2205 DSS butt joint shows higher tensile properties than both BM and GTAWed joints. The Ys, UTS, and E% of the FSWed joint are enhanced over BM by 65%, 33%, and 54%, and over the GTAW weldment by 21%, 41%, and 66%, respectively.

## Figures and Tables

**Table 1:** Chemical composition of the 2205 DSS base material (in wt.%).

Cr	Ni	Mo	Mn	Si	N	C	P	S	Fe
22.43	5.74	3.15	1.51	0.38	0.17	0.015	0.025	0.001	Bal.

**Table 2:** Chemical composition of the E2209 filler rod (in wt.%).

Cr	Ni	Mo	Mn	Si	N	C	P	S	Cu	Fe
22.10	9.42	3.03	0.76	0.86	0.14	0.022	0.015	0.016	0.05	Bal.

**Table 3:** GTAW welding parameters.

Pass	Filler rod	Ampere (A)	Volt (V)	Travel Speed (mm/min)	Heat Input (kJ/mm)
1st	ER2209	116	10	90	0.733
2nd		159	13	90	1.378
3rd		155	13.5	81	1.550
Cap1		150	14.5	76	1.717
Cap2		116	10	135	0.516
